# Supplementary material for: Field-induced compensation of magnetic exchange as the possible origin of reentrant superconductivity in UTe2
Source: Nat Commun. 2024 Jan 2;15:37. doi: 10.1038/s41467-023-44183-1 (PMC10761692; doi:10.1038/s41467-023-44183-1)
Supplement: Supplementary file 1 — Supplementary Information [file 41467_2023_44183_MOESM1_ESM.pdf]

SUPPLEMENTARY IMFORMATION:

Field-induced compensation of magnetic exchange as the possible origin of reentrant superconductivity in UTe_2

Toni Helm[1,2,*], Motoi Kimata[3], Kenta Sudo[3], Atsuhiko Miyata[1],
Julia Stirnat[1,4], Tobias Förster[1], Jacob Hornung[1,4],
Markus König[2], Ilya Sheikin[5], Alexandre Pourret[6],Gerard Lapertot[6],
Dai Aoki[7], Georg Knebel[6], Joachim Wosnitza[1,4], Jean-Pascal Brison[6]

[1]Hochfeld-Magnetlabor Dresden (HLD-EMFL) and Würzburg-Dresden Cluster of Excellence ct.qmat, Helmholtz-Zentrum Dresden-Rossendorf, 01328 Dresden, Germany

[2]Max Planck Institute for Chemical Physics of Solids, 01187 Dresden, Germany

[3]Institute for Materials Research, Tohoku University, Sendai, Miyagi, 980-8577, Japan

[4]Institut für Festkörper- und Materialphysik, Technische Universität Dresden, 01062 Dresden, Germany

[5]Laboratoire National des Champs Magnétiques Intenses (LNCMI-EMFL), CNRS, UGA, 38042 Grenoble, France

[6]Univ. Grenoble Alpes, CEA, Grenoble-INP, IRIG, PHELIQS, 38000 Grenoble, France

[7]Institute for Materials Research, Tohoku University, Oarai, Ibaraki, 311-1313, Japan

*Corresponding author: **t.helm@hzdr.de**

(Dated: December 1, 2023)

SUPPLEMENTARY NOTE 1: TORQUE MAGNETOMETRY RESULTS

In Fig. S1, we present the torque data recorded at 0.7 and 1.5 K in pulsed magnetic fields up to 70 T. We applied a constant offset to the curves at different fixed angles for better visibility. The transition-field values, presented in the main text in Fig 1a, are marked at half the height of the jump in τ. In figures (c) and (d) we show the same torque data plotted against the tilt angle θ, for selected (marked) field values between 20 and 65 T in 5 T steps.

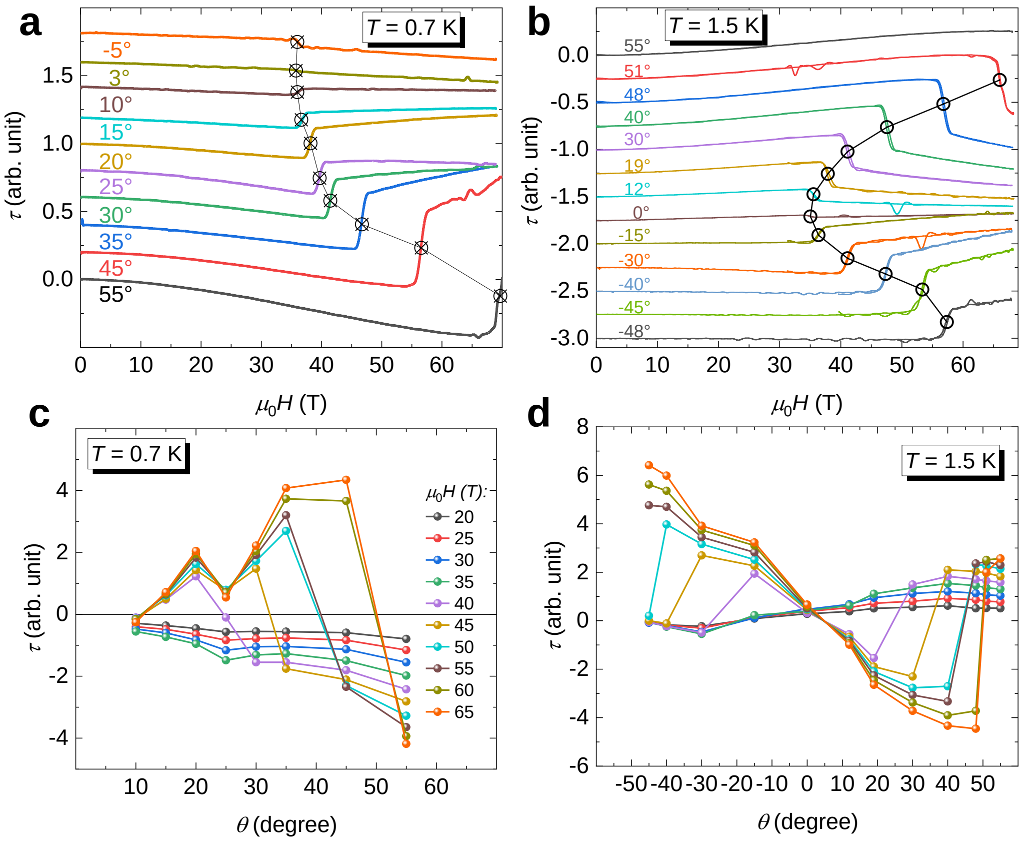

FIG. S1. **Magnetic torque of UTe_2.** (a), (b) Magnetic torque recorded at 0.7 K and 1.5 K for various angles in the (b, c) plane. The tilt angle $\theta = 0°$ corresponds to $H \parallel b$. (c), (d) Amplitude of the torque determined at fixed field values plotted against the angular tilt. Note: The color-code for the field values is the same in both figures. The asymmetry between positive- and negative-angle curves is caused by the asymmetric deflection stiffness of the piezoresistive microcantilever.

SUPPLEMENTARY NOTE 2: BASIC TRANSPORT CHARACTERIZATION

The FIB microfabrication process introduces an amorphized layer enriched with implanted Ga atoms (thickness: $\sim 20\,\mathrm{nm}$). Hence, the standard first step in characterization of a FIB-cut (An example image is shown in Fig. S2a) device is to test the zero-field resistivity. In Fig. S2b we present a comparison of the a-axis resistivity for the bulk single crystal and device #1. Apparently the overall residual-resistance ratio and T_c has not been altered by the fabrication procedure. Slight deviations may originate from differences in the

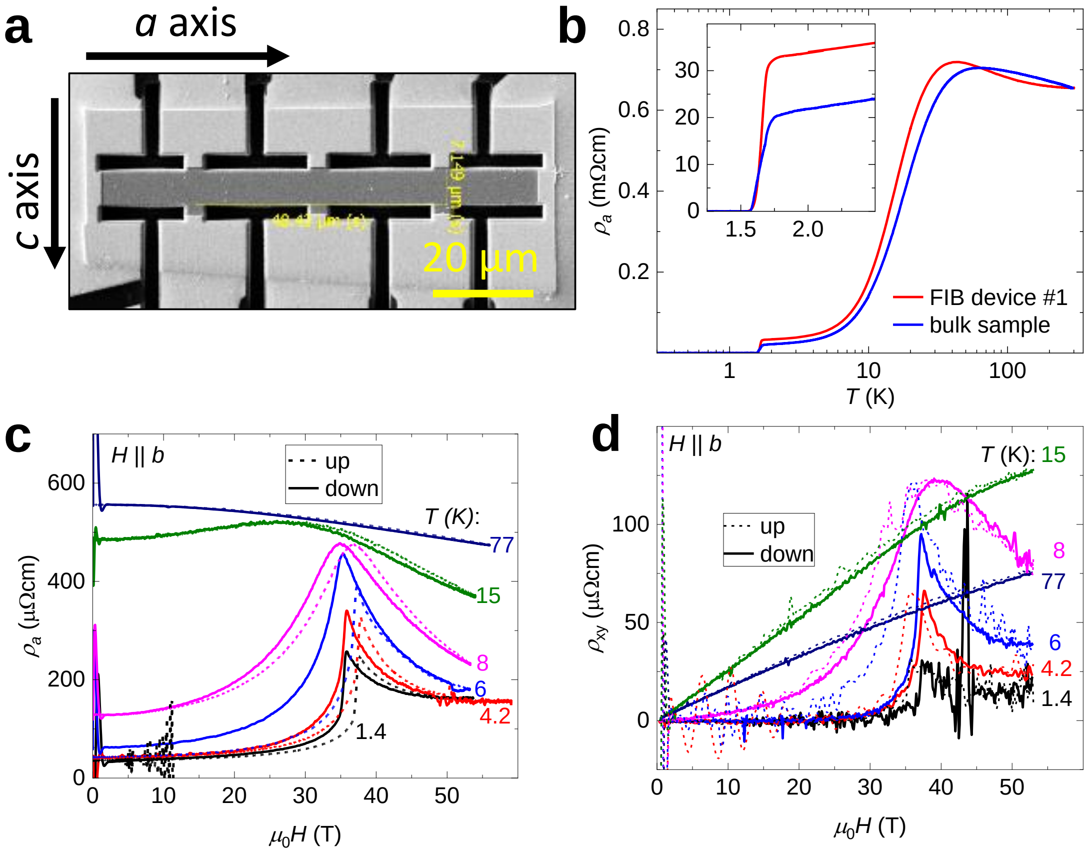

FIG. S2. **Basic transport characterization of UTe_2 FIB devices.** (a) SEM image of Hall-bar device #3. (b) Comparison of zero-field a-axis resistivity for a bulk Hall-bar device and the FIB-microstructured device #1, with dimension of $(2\times0.5\times0.1)\,\mathrm{mm}^3$ and $(13\times5\times2)\,\mu\mathrm{m}^3$, respectively. The inset highlights the matching T_c. (c) Magnetoresistivity and (d) Hall resistivity of device #3 recorded in a 60 T short-pulse (25 ms) magnet at various temperatures. These results match nicely with data published previously for bulk samples [1], confirming that FIB treatment does not alter the transport properties.

homogeneity of stress induced by the sample substrate.

Figure S2a shows an scanning-electron-microscope (SEM) image of device #3. The measured $\rho_a(H)$ and $\rho_{xy}(H)$ reproduce results previously reported for bulk samples to temperatures of up to 77 K (Figs. S2c and d) [1]. The measurements for this device were carried out in a pulsed magnet with a shorter pulse duration ($\sim$ 25 ms). This is why there is a significant hysteresis between the up- (dotted) and the down-sweep (solid) curves. Device #3, however, did not survive transfer in air to perform additional experiments. The air sensitivity of UTe_2 may be responsible for a fast deterioration of the contacts. Consequently, the devices labeled with #1 and #2 were sealed by enclosing them with epoxy, for further details see the Methods section.

SUPPLEMENTARY NOTE 3: ADDITIONAL HIGH-FIELD MAGNETORESISTIVITY DATA FOR DEVICE #2

In Fig. S3 we provide data recorded for device #2 at various angles and temperatures. The critical-field values presented in Fig. 3c and d were extracted from this data set.

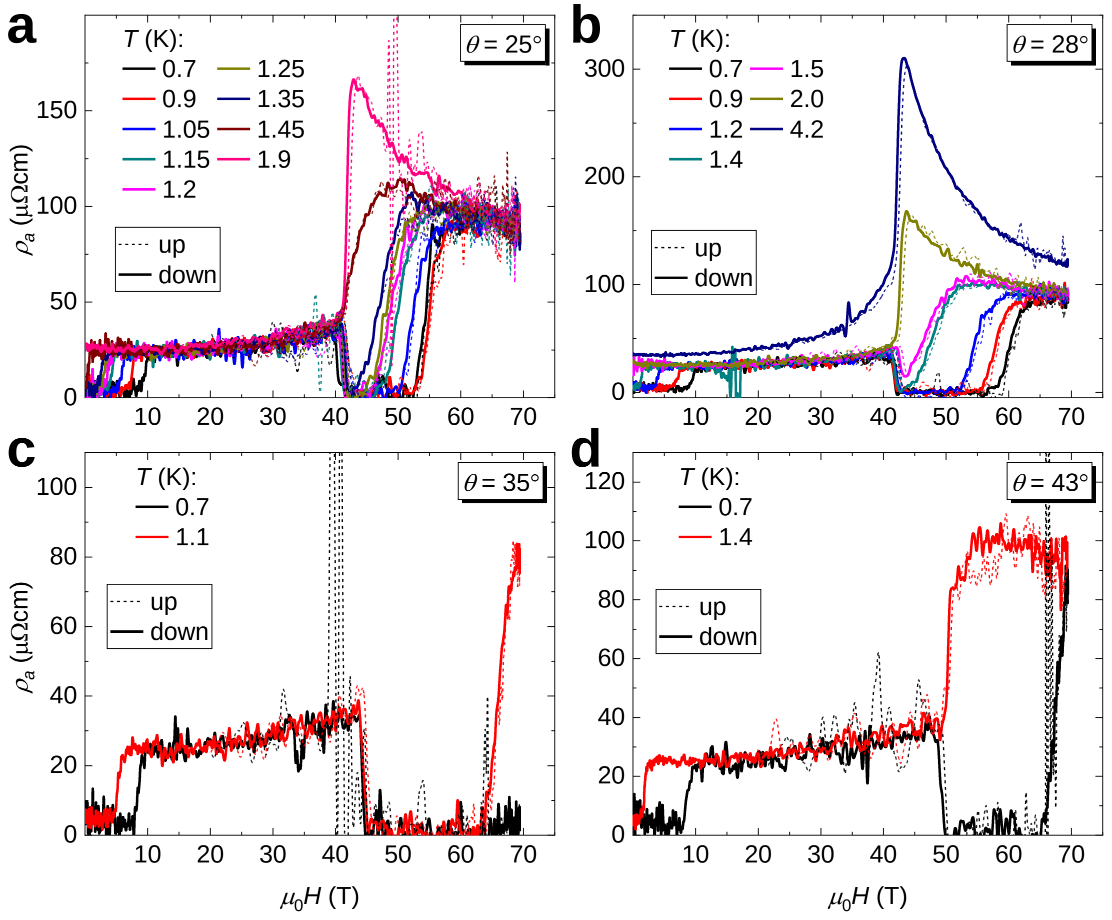

FIG. S3. **Magnetoresistivity of UTe_2 recorded for device #2 at four fixed angles** with applied current of 100 μA in a 70 T pulsed magnet. Up and down seeps are marked by dotted and solid lines, respectively.

Anisotropy of H_{c2} in the high- and low-field superconducting phases

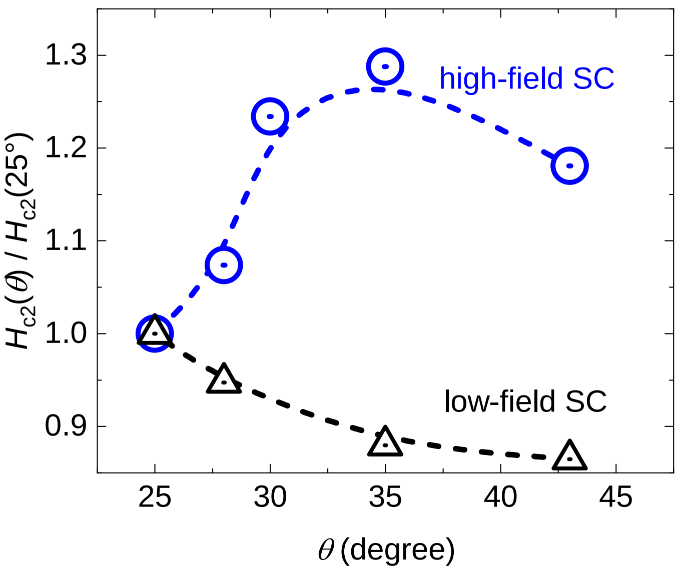

FIG. S4. **Anisotropy of H_{c2} in the hfSC and lfSC phases.** Normalized upper-critical-field values at absolute zero in the hfSC and lfSC phases extracted from in Fig. 2e. The data were normalized to the respective H_{c2} values at 25°. Dashed lines are guides to the eye.

SUPPLEMENTARY NOTE 4: ANOMALOUS HALL EFFECT AT TILT ANGLE

In Fig. S5, we present the analysis of the field-dependent Hall effect (first column) following the formalism previously applied to $4f$ and $5f$ compounds at low temperatures [2, 3]. Under the assumption that skew scattering (extrinsic) is the dominating contribution to the anomalous Hall effect of UTe_2, the Hall resistivity may be proportional to the square of the longitudinal resistivity multiplied by the magnetization in the coherent regime at low temperature [2, 4]. We plot the scaled Hall resistivity ρ_{xy}/H against the product $\rho_{xx}^2 M/H$ scaled by magnetic field (middle panels in Fig. S5). The data sets, both at low fields ($0\,\mathrm{T} \leq \mu_0 H \leq 32\,\mathrm{T}$) in the second column and in the high-field range ($45.5\,\mathrm{T} \leq \mu_0 H \leq 65\,\mathrm{T}$) in the third column, exhibit linear dependencies. In order to account for the magnetization at $\theta \approx 30°$, we included previously published data from Ran et al. and Miyake et al. [5, 6]. Compared to previous results obtained for $H \parallel b$, the data show a similar linearity indicative for an extrinsic anomalous Hall effect (AHE) component associated with skew scattering From this analysis, the normal Hall coefficient R_0 can be estimated from the intercept, indeed $\rho_{xy}/H = R_0 + \rho_{xx}^2 M/H$. At the lowest temperature (0.6 K), the resulting value changes from $0.06\,\mu\Omega\mathrm{cm}$ to about $0.1\,\mu\Omega\mathrm{cm}$ upon transitioning from below to above H_m. This corresponds to a jump by roughly factor of two. In comparison, ρ_{xy} changes by a factor of 10 for $H \parallel b$ [3]. The latter result was associated with a significant Fermi-surface reconstruction at H_m due to a drastic reduction of the carrier concentration. However, other quantities such as the A coefficient in the temperature-dependent resistivity [1] or the Sommerfeld coefficient (predicted from magnetization results [6]) do not exhibit similar dramatic jumps. Therefore, the result suggests an additional intrinsic AHE component (associated with a topological Berry-curvature contribution), which is independent of the scattering time τ and, thus, disregarded by the analysis described above. Another point, in favor of an enhanced intrinsic anomalous-Hall component, would be that the overall Hall conductivity above H_m is of the order of $10^4 - 10^5 (\Omega\mathrm{cm})^{-1}$. According to what has been empirically determined for various materials (see the review by Nagaosa [4]), this is assumed to be the "good-metal" regime, mainly dominated by intrinsic contributions in the Hall effect.

Such intrinsic contributions may be described in terms of the Berry curvature formalism and can arise from various origins, e.g.:

- Avoided band crossings in the spin-polarized band structure close to or at the Fermi

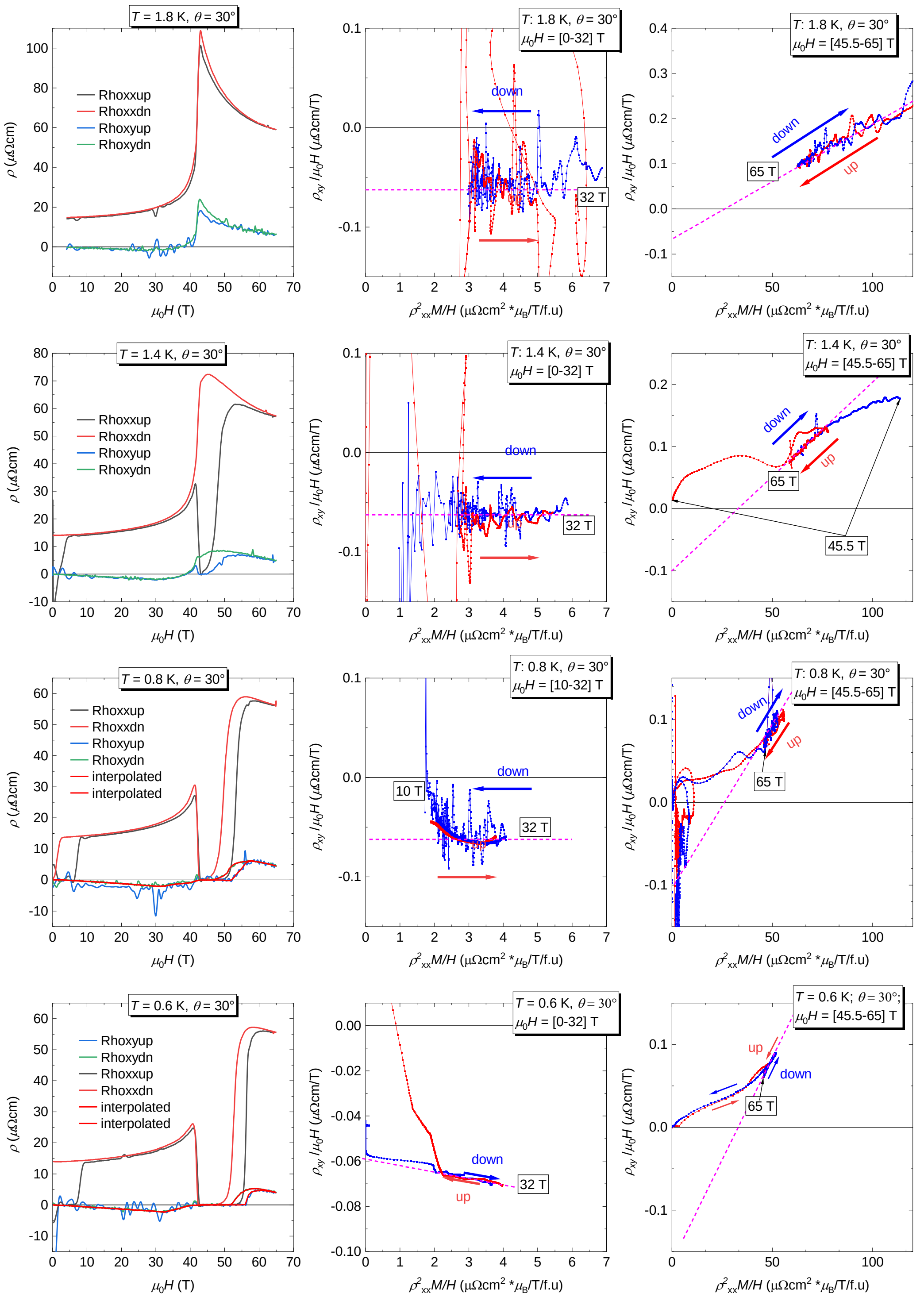

FIG. S5. **Analysis of the AHE at $\theta = 30^\circ$ following Refs. [2, 3].** Left column: data set recorded for device #1 at $\theta = 30^\circ$ with $I = 500\,\mu$A for $T = 0.6$, 0.8, 1.4, and 1.8 K, respectively. Middle and right column: ρ_{xy}/H against $\rho_{xx}^2 M/H$ in the low and high-field range, respectively. Note: Dashed magenta lines are linear fits used to extract R_0 from the intercepts.

edge [7],

- Complex magnetic order involving spin-chirality scattering effects that may cause an additional transverse anomalous velocity [8].

We believe that this is an important finding, as we demonstrate here that exactly this enhanced intrinsic Hall component seems to get drastically suppressed at the angular range where the hfSC phase emerges. Interestingly, Weyl physics has already been proposed to be of importance in UTe_2 [9]. In order to estimate the Berry-curvature effect in momentum space, detailed information on the Fermi surface in the high-field phase are required. As first magnetic quantum oscillations have already been reported [10, 11], it may become possible in the near future to obtain details about the effect of H_m on the band structure of UTe_2.

The general analyses of the Hall effect is complicated by the appearance of metamagnetism in UTe_2, the lack of detailed information about the magnetic structure and its effects on the band structure of UTe_2. In order to discriminate the orbital from the anomalous coefficient an approach would be to study the high-field Hall effect in the field range where the magnetism is saturated so that the AHE contribution remains constant. As the magnetization of UTe_2 does not saturate above H_m up to 70 T such an approach could not be applied in our case.

SUPPLEMENTARY NOTE 5: AN ATTEMPT TO FIT H_{c2} IN THE HFSC PHASE ASSUMING JACCARINO-PETER COMPENSATION.

Recently, Rosuel et al. [12] showed that the field-reinforced sc phase close to (below) H_m for $H \parallel b$ can be described by a field-controlled strong-coupling constant $\lambda(H)$ in the strong-coupling regime. The model relies on a strong-coupling calculation of H_{c2} for s-wave superconductors, including both orbital and paramagnetic effects. The effect of spin-triplet pairing is "mimicked" by suppressing the paramagnetic effect. The parameters of the model for the determination of the critical temperature are a typical energy scale of the fluctuations responsible for the pairing (Ω), the pairing strength λ, and a screened Coulomb repulsion (μ^*). The spectral density of interactions is defined in a minimal form [13]:

$$\alpha^2 F(\omega) = \frac{\lambda\Omega}{2}\delta(\omega - \Omega). \tag{S1}$$

T_c and the H_{c2} are derived from the solution of the resulting Eliashberg equations. This also yields a renormalization of the bare averaged Fermi velocity ,$\langle v_F \rangle$, that controls H_{c2} in a given direction (see Appendix of Ref. [12]).

The field dependence of λ is required to account for the reinforcement of H_{c2} for $H \parallel b$. It is deduced from the comparison between the experimental data and calculations for different values of λ, which is the most sensitive parameter of the model (Ω and μ^* are chosen to remain field independent).

Two scenarios were proposed in Ref. [12] for the high-field field-reinforced phase ($H \parallel b$): Either a spin-triplet ESP state for the field-reinforced phase, hence, with no paramagnetic limit at all, or a (counter-intuitive) spin-singlet phase, possibly linked to antiferromagnetic fluctuations that develop upon approaching H_m. In the second case, the enhancement of the paramagnetic limit, due to the increase of the λ, plays a major role for the control of H_{c2} in the field-reinforced phase. Support for this scenario stems from the strong broadening of the specific-heat anomaly observed in the field-reinforced phase and from the angular dependence of this broadening [12].

We extend this model to finite tilt angles by including two simple hypotheses: Following Ref. [12], $\lambda(H)$ in the field-reinforced phase follows the angle-dependence of H_m, hence, $\lambda(H/H_m(\theta))$, with $H_m \propto 1/\cos\theta$). For $\langle v_F \rangle$ controlling H_{c2}, if $\bar{v}_b^0$ and $\bar{v}_c^0$ are those for fields along the b and c axis, respectively, at an angle θ between the two we use:

$$\langle v_F(\theta) \rangle = \sqrt{\left(\bar{v}_b^0 \cos\theta\right)^2 + \left(\bar{v}_c^0 \sin\theta\right)^2}. \tag{S2}$$

Here, we assume an isotropic Pauli limit, hence, no angular dependence of g (with an absolute value of $g = 0$ in the ESP spin-triplet case or $g = 2$ in the spin-singlet case).

We apply this model to the hfSC phase using the same values of Ω, μ^*, $\bar{v}_b^0$, and $\bar{v}_c^0$ for all scenarios, consistent with Ref. [12]. Therefore, we neglect any effects related to possible changes of the Fermi surface [14] or the characteristic fluctuation energy at H_m. However, we assume that the compensation effect between H and H_{ex}, together with the "distance" of H from H_m lead to a new field dependence of λ. For the scenario of spin-singlet pairing this will change the paramagnetic limit as well.

This last point requires a determination of the compensation as a function of H and θ. At the mean-field level, as mentioned in the main text, H_{ex} depends crucially on the H dependence of both the longitudinal and transverse magnetization: $H_{ex} = J_c < M_c >$

$\hat{c} + J_b < M_b > \hat{b}$, with the anisotropic exchange constants J_c and J_b and the magnetization components $< M_c >$ and $< M_b >$ along the c and b axes, respectively. Presently, the dependence of the magnetization on H is unknown: Our magnetic-torque measurement and the large jump observed at H_m indicate that the magnetization jump is not purely longitudinal. It is even likely that $< M_b >$ remains close to its value for $H \parallel b$ at H_m, which is in agreement with the angle dependence of H_m, following $1/\cos\theta$. However, the large jump in $M(H)$ at H_m observed for finite tilt angle in previous works [5, 15] is also indicative of a significant component along c, likely to grow further for fields above H_m. Additional experiments are required to determine the details of this dependence.

Hence, we have limited the evaluation of this scenario to the H_c2 data set at $\theta = 35°$, where the almost complete suppression of the Hall angle at 70 T suggests a very good compensation of H by H_ex. Furthermore, we assume that above H_m, up to 70 T, H_ex (linked to the magnetization jump) remains constant, equal to -70 T, and collinear with H. In UTe_2, the crystallographic orientation [011] seems specific in many respects: The (011) plane is a natural cleavage plane of single crystals of UTe_2; It is associated with a Yamaji magic angle related to the warping of the Fermi Surface; It becomes the new direction for the tetragonal c axis after the recently observed pressure-induced structural transition [16]. Microscopic models might explain if its importance for the magnetic properties of UTe_2 is coincidental. Perfect compensation at 70 T means that at this field, H_c2 should be completely limited by the orbital effect. With $\langle v_\mathrm{F}(\theta)\rangle$ determined as in Eq. S2, the only parameter left to be adjusted is the value of λ at this field. Fig. S6 shows the data of UTe_2 at 35° for both in the lfSC and hfSC phases, and the orbital H_c2 calculated for an H-independent value of $\lambda = 1.58$ (dashed purple line).

In Fig. S6, we also show the result of the same calculation with $\lambda = 1.58$ including the paramagnetic limit (solid green line), controlled by $g\mu_\mathrm{B}(H - H_\mathrm{ex})$, where H_ex is zero below H_m and -70 T above H_m. The reentrant hfSC phase then can emerge above H_m thanks to the compensation by H_ex. This is clearly visible for both λ values shown. For the H_c2 value closest to H_m we found $\lambda = 1.94$ (blue solid line in Fig. S6). Below H_m, we find that the maximum of H_c2 for both λ values, namely $H_\mathrm{c2}(0) \approx 17$ T and $H_\mathrm{c2}(0) \approx 25$ T, respectively, lie below the H values necessary to reach the corresponding $\lambda(H)$ (see Fig. 4b in the main text: $H(\lambda = 1.58) \approx 22$ T or $H(\lambda = 1.94) \approx 32$ T. As a consequence, SC is suppressed by the paramagnetic limit. The reason is that H_m increases approximately with $1/\cos(\theta)$ and below

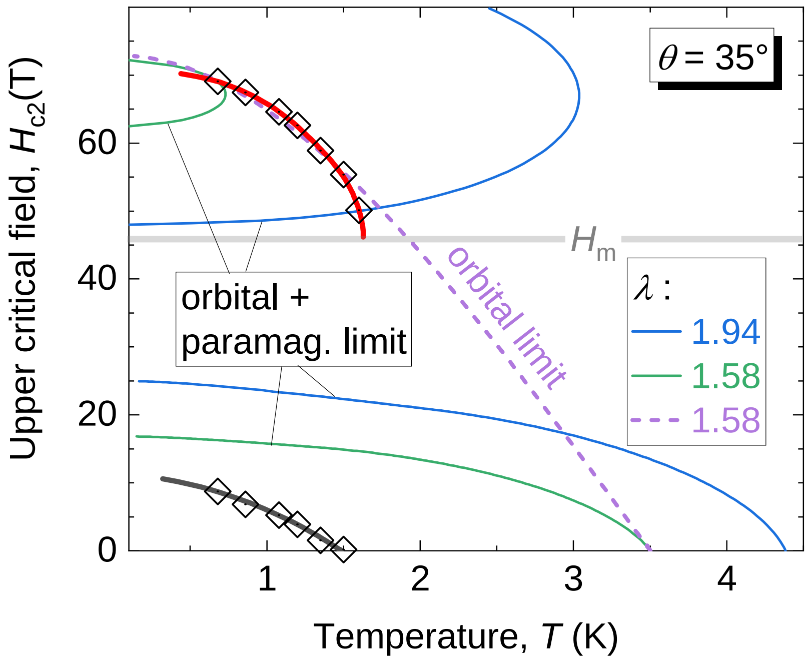

FIG. S6. $H_{c2}(T)$ **including the Jaccarino-Peter compensation effect** [17]**.** The black diamonds are H_{c2} values in the LF and hfSC phases as determined by experiment for $\theta = 35°$. Black solid line is an orbital fit using $\lambda(H)$ (presented in the main text in Fig. 4b) for the low-field phase, and parameters for the Fermi velocity along b and c axes taken from Ref. [12]. The purple dashed line is a fit in the pure orbital limit with a constant value of $\lambda = 1.584$, and the same bare Fermi velocity as in the LF phase. Green and blue solid lines are fits including orbital and paramagnetic limitation with two different λ values as indicated, both below H_m (without H_{ex}, i.e., no compensation) and above H_m, where $H_{ex} = -70\,$T (collinear with H) partly compensates the external field. The red solid line is the resulting fit using the $\lambda(H)$ (presented in the main text in Fig. 4b) for $H \geq H_m$.

H_m the H-induced increase of $\lambda(H/H_m)$ at 35° is too small to prevent the paramagnetic limit from suppressing the superconducting state. The complete $\lambda(H)$ curve above H_m at 35° in Fig. 4b is deduced from a smooth fit of the values of λ required to reproduce the data within both scenarios (Jaccarino-Peter compensation shown in Fig. S6 and pure orbital limitation shown in Fig. 2e). The overall H dependence of λ resembles that of the specific heat (C_p/T) in the normal state around H_m for field along the b axis [12]. It differs, however, from the positive jump that was proposed from analysis of the magnetization by the Clausius-Clapeyron relations [15]. As explained in Ref. [12], C_p/T in UTe_2 even at temperatures as

low as 1.8 K is rather complex and consists more contributions than that of the Sommerfeld coefficient. Thus, it is difficult to draw a direct connection between the H dependence of λ and that of C_p/T (through the mass renormalization induced by pairing interactions). Moreover, at H_{m}, many phenomena can come into play, e.g, a change of the band structure (Fermi-surface topology), which are not directly related to λ.

REFERENCES

[1] Knafo, W. et al. Magnetic-Field-Induced Phenomena in the Paramagnetic Superconductor UTe_2. J. Phys. Soc. Jpn. **88**, 063705 (2019). URL https://doi.org/10.7566/JPSJ.88.063705.

[2] Yamada, K., Kontani, H., Kohno, H. & Inagaki, S. Anomalous Hall Coefficient in Heavy Electron Systems. Progress of Theoretical Physics **89**, 1155–1166 (1993). URL https://doi.org/10.1143/ptp/89.6.1155.

[3] Niu, Q. et al. Evidence of Fermi surface reconstruction at the metamagnetic transition of the strongly correlated superconductor UTe_2. Phys. Rev. Res. **2**, 033179 (2020). URL https://link.aps.org/doi/10.1103/PhysRevResearch.2.033179.

[4] Nagaosa, N., Sinova, J., Onoda, S., MacDonald, A. H. & Ong, N. P. Anomalous Hall effect. Rev. Mod. Phys. **82**, 1539–1592 (2010). URL https://link.aps.org/doi/10.1103/RevModPhys.82.1539.

[5] Ran, S. et al. Extreme magnetic field-boosted superconductivity. Nat. Phys. **15**, 1250–1254 (2019). URL https://doi.org/10.1038/s41567-019-0670-x.

[6] Miyake, A. et al. Metamagnetic Transition in Heavy Fermion Superconductor UTe_2. J. Phys. Soc. Jpn. **88**, 063706 (2019). URL https://doi.org/10.7566/JPSJ.88.063706.

[7] Burkov, A. A. Anomalous Hall Effect in Weyl Metals. Phys. Rev. Lett. **113**, 187202 (2014). URL https://link.aps.org/doi/10.1103/PhysRevLett.113.187202.

[8] Fujishiro, Y. et al. Giant anomalous hall effect from spin-chirality scattering in a chiral magnet. Nat. Commun. **12**, 317 (2021). URL https://doi.org/10.1038/s41467-020-20384-w.

[9] Shishidou, T., Suh, H. G., Brydon, P. M. R., Weinert, M. & Agterberg, D. F. Topological

band and superconductivity in UTe_2. Phys. Rev. B **103**, 104504 (2021). URL https://link.aps.org/doi/10.1103/PhysRevB.103.104504.

[10] Aoki, D. et al. First Observation of de Haas-van Alphen Effect and Fermi Surfaces in Unconventional Superconductor UTe_2 (2022). URL https://arxiv.org/abs/2206.01363.

[11] Eaton, A. G. et al. Quasi-2D Fermi surface in the anomalous superconductor UTe_2 (2023). URL https://arxiv.org/abs/2302.04758.

[12] Rosuel, A. et al. Field-Induced Tuning of the Pairing State in a Superconductor. Phys. Rev. X **13**, 011022 (2023). URL https://link.aps.org/doi/10.1103/PhysRevX.13.011022.

[13] Bulaevskii, L. N., Dolgov, O. V. & Ptitsyn, M. O. Properties of strong-coupled superconductors. Phys. Rev. B **38**, 11290–11295 (1988). URL https://link.aps.org/doi/10.1103/PhysRevB.38.11290.

[14] Niu, Q. et al. Fermi-Surface Instability in the Heavy-Fermion Superconductor UTe_2. Phys. Rev. Lett. **124**, 086601 (2020). URL https://link.aps.org/doi/10.1103/PhysRevLett.124.086601.

[15] Miyake, A. et al. Enhancement and Discontinuity of Effective Mass through the First-Order Metamagnetic Transition in UTe_2. J. Phys. Soc. Jpn. **90**, 103702 (2021). URL https://doi.org/10.7566/JPSJ.90.103702.

[16] Honda, F. et al. Pressure-induced Structural Phase Transition and New Superconducting Phase in UTe_2. Journal of the Physical Society of Japan **92**, 044702 (2023). URL https://doi.org/10.7566/JPSJ.92.044702. https://doi.org/10.7566/JPSJ.92.044702.

[17] Jaccarino, V. & Peter, M. Ultra-high-field superconductivity. Phys. Rev. Lett. **9**, 290–292 (1962). URL https://link.aps.org/doi/10.1103/PhysRevLett.9.290.
